# Enhancing physiological performance and quality traits of lettuce (*Lactuca sativa* L. cv. 'Batavia') through red–blue light ratio and γ-aminobutyric acid interplay

**DOI:** 10.1186/s12870-026-08493-y

**Published:** 2026-03-16

**Authors:** Morteza Karimi, Shima Mirzaei, Sasan Aliniaeifard, Seyyed Hassan Mousavi, Saeid Hazrati, Nazim S. Gruda

**Affiliations:** 1 Department of Horticulture, Faculty of Agricultural Technology (Aburaihan), University of Tehran, Tehran, 14395-547 Iran; 2 Greenhouse and Controlled Environments Research Institute, Horticultural Sciences Research Institute, Agricultural Research, Education and Extension Organization, Karaj, Alborz 14176 Iran; 3 Department of Agronomy and Plant Breeding, Faculty of Agriculture, Azarbaijan Shahid Madani University, Tabriz, 5375171379 Iran; 4 Department of Horticultural Science, INRES-Institute of Crop Science and Resource Conservation, University of Bonn, Bonn, 53121 Germany

**Keywords:** Biomass Partitioning, Chlorophyll Fluorescence, Controlled environment agriculture, LED Spectrum Optimization

## Abstract

**Background:**

The integration of tailored light spectra and γ-aminobutyric acid (GABA) supplementation represents a promising strategy for improving crop yield in controlled environment agriculture (CEA). This study investigated the combined effects of Red (R):Blue (B) light ratios (RB: 90:10, 80:20, and 70:30) and 25 μmol L^−1^ GABA supplementation (G25) compared with the control (G0) on growth, chlorophyll fluorescence, and quality of *Lactuca sativa* L. cv. Batavia in a vertical farming system.

**Results:**

Plant morphology was modulated by light spectrum and GABA. The RB:80:20 + G25 treatment significantly increased leaf anthocyanin content by 31.3%, leaf area by 15.4%, total fresh weight by 20%, and soluble carbohydrate content by 26.3% compared to the control plants. In contrast, the RB:70:30 treatment directed plant growth toward the roots. However, GABA application increased biomass reallocation toward the shoots by 60%. GABA application reduced the excess excitation energy (DI_0_/RC) by 11.76%, highlighting its role in improving light use efficiency. The highest F_j_ and F_0_ values were recorded in RB:90:10 + G25 and RB:80:20 + G25 treatments, respectively. This indicates the greater absorption range of R light by leaf chlorophylls, its impact on plant photosynthesis and the importance of GABA in modulating light transmission in the O-J phase. Sensory evaluation confirmed that RB:80:20 + G25 scored the highest for visual quality and taste.

**Conclusions:**

These findings suggest that precise combinations of R:B light ratios and GABA supplementation can simultaneously enhance and optimize both physiological performance and consumer-oriented quality traits in lettuce. Future research should investigate the molecular mechanisms underlying these interactions and their potential application to other leafy crops in CEA systems.

**Graphical Abstract:**

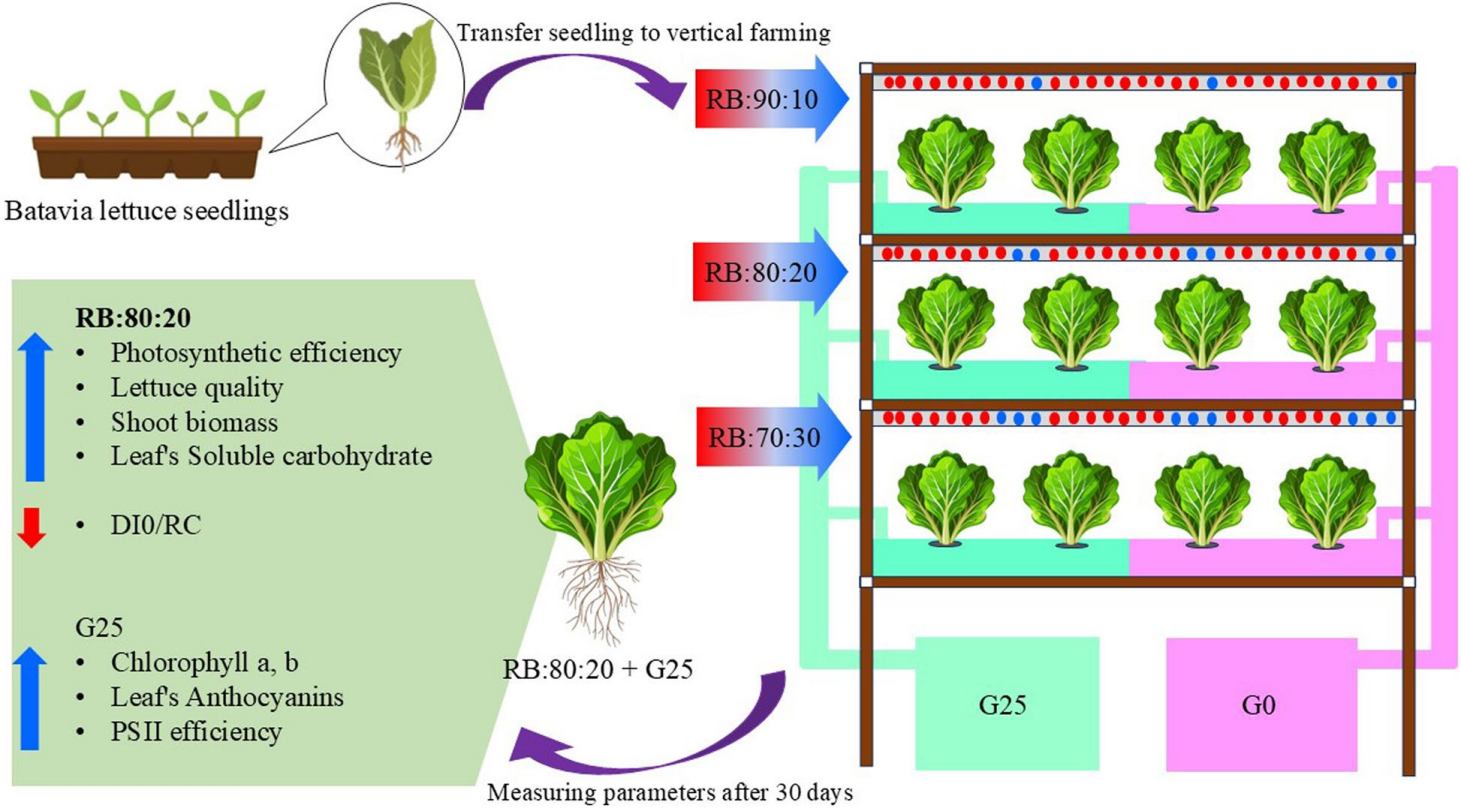

**Supplementary Information:**

The online version contains supplementary material available at 10.1186/s12870-026-08493-y.

## Background

Controlled environment agriculture (CEA) is an innovative technique that uses artificial lighting and precise nutrient management to optimize plant growth [1, 2], and address current agricultural challenges and climate change effectively [3]. Among the environmental factors in CEA, optimizing the light spectrum plays a crucial role in determining plant physiological responses and crop quality [4]. Photosynthetically active radiation (PAR) refers to the visible light wavelengths between 400 and 700 nm (nm) that can be absorbed by chlorophyll and activate the electron transport chain in chloroplasts [5]. The specific wavelengths within the PAR spectrum have different effects on plant physiological and developmental responses [6]. Two absorption peaks in the red (R) (600–700 nm) and blue (B) (400–500 nm) regions of the chlorophyll quantum yield curve significantly impact plant photosynthesis [7].

Previous studies have demonstrated that monochromatic light can cause physiological disorders in plants. While R light increases photosynthesis and biomass accumulation, its use alone can cause “Red Light Syndrome”, which disrupts chlorophyll morphology and content [8]. Additionally, B light enhances stress adaptation, phototropism, and photomorphogenesis, but it can also reduce leaf area (LA) and root growth due to excessive energy and an imbalance between phytochromes and cryptochromes [9]. However, combining R and B light mitigates these disadvantages, improving photosynthetic efficiency, chlorophyll synthesis and biomass [10]. Optimizing light quality, particularly the R and B spectra, is essential for crop production in CEA [11]. A light spectrum of RB:70:3070 enhance the growth of *Valeriana officinalis* L. [12]., and *Crocus sativus* L. [13] compared to monochromatic R and B light. A light spectrum of RB:50:50 increased the quality and antioxidant content of *Eruca sativa* L. plants compared to those grown under other light spectra (R:G:B 40:20:40 and white) [14]. Light combinations of R and B with ratios of 70:30 and 50:50 led to maximum dry weight (DW) and photochemical efficiency of Photosystem II (PSII) in *Cucumis melo* L. [15], and *Cucumis sativus* [16] respectively. Additionally, a RB:75:25 light combination increased the chlorophyll content, yield and water content of *Ocimum basilicum* L. [17].

The combination of R and B light is essential for crop growth and photosynthesis because it corresponds to the peak of chlorophyll absorption. However, R light is absorbed more efficiently by chlorophyll and drives photosynthesis more effectively than B light [18]. R light also constitutes a greater percentage of the total spectrum [19]. Furthermore, high R light can significantly accelerate photosynthesis and increase carbohydrate synthesis and biomass yield in *Coriandrum sativum* L. [20]. The RB:80:20 spectrum increased antioxidant enzyme activity, while the RB:70:30 spectrum increased the accumulation of soluble sugars and amino acids in *Catharanthus roseus* [21]. Different species respond differently to varying ratios of R and B light [22]. For example, an RB:70:30 light spectrum treatment increased the concentration of vitamin C, soluble carbohydrates, and anthocyanins in *Brassica rapa* subsp. chinensis cv. 'Green Fortune', whereas an RB:80:20 light spectrum treatment suppressed these traits in *Brassica rapa* subsp. chinensis cv. 'Red Choi'. These findings suggest that even different cultivars of the same species may respond differently to various light quality combinations [23].Therefore, it is essential to adjust the R:B light ratio appropriately to maximize plant growth in controlled environments depending on the plant genotype [24].

Recent research suggests that, while precise R:B light ratios optimize photosynthesis, GABA supplementation can enhance plant performance further by reducing the inhibition of photosynthesis induced by stress and improving overall growth resilience [25]. GABA, a four-carbon non-protein amino acid, plays a pivotal role in plant growth, development and stress responses [26]. Beyond stress responses, GABA promotes growth under non-stress conditions by improving nitrogen assimilation [27], stimulating root development through interactions with the auxin pathway [28], and enhancing postharvest quality via stomatal regulation [29]. The effects of GABA are modulated by light conditions; recent studies have shown that combining it with optimized R:B light spectra enhance lettuce growth compared to monochromatic treatments, likely through the coordinated regulation of stomatal conductance and photosynthetic enzymes [30, 31]. This interaction occurs through cryptochrome mediated B light responses. It also shifts the effect of R light on glutamate metabolism in maize leaves from GDH to the GABA shunt. This enables GABA to maintain the tricarboxylic acid (TCA) cycle during light induced metabolic changes. These findings highlight the potential of GABA as a strategic tool for improving crop performance in controlled environment agriculture systems [32, 33].

Lettuce (*Lactuca sativa* L.) is a widely cultivated leafy vegetable renowned for its nutritional value, which includes high levels of vitamins, minerals and dietary fiber [34]. Its rapid growth cycle, adaptability to various environmental conditions, suitable height and short harvest period make it an ideal model crop for scientific research and cultivation in CEA [35]. It is a high yield crop that can be produced consistently throughout the year in CEA equipped with artificial lighting [36], reducing the impact of external factors such as the season, climate constraints, pests, and diseases [37]. Using artificial light, particularly the R and B spectra [28], and GABA [38], has been shown to optimize lettuce growth and quality. Studies have demonstrated that particular wavelengths can enhance photosynthesis, biomass accumulation, and nutrient content in lettuce plants [39].

Despite extensive research on the effects of light spectra on plant growth, the optimal R:B light ratio for enhanced growth and quality in Batavia lettuce, especially in CEA systems, has not yet been fully defined. More importantly, few studies have examined how metabolic regulators, such as GABA, interact with light spectrum manipulation to optimize plant responses. While the independent effects of R:B light ratio and GABA supplementation have been studied in other crops, the synergistic interaction between these two factors has not been adequately investigated, particularly in the context of Batavia lettuce. This gap in knowledge is significant because Batavia lettuce exhibits unique physiological and morphological characteristics that may respond differently to R:B light ratios and GABA treatments compared to other cultivars. Furthermore, the increasing demand for high quality, resource-efficient products in CEA highlights the need for innovative strategies that can maximize yield and nutritional quality while minimizing resource input [40].

This study is novel in that it examines the previously unexplored synergistic interactions between R:B light ratios and exogenous GABA on photosynthetic performance in Batavia lettuce. Prior research has only investigated these factors in isolation. The aim was to investigate how specific R:B light ratios interactively modulate GABA mediated responses in PSII efficiency, chlorophyll biosynthesis, biomass accumulation and quality traits.

We hypothesize that optimized R:B light ratios will enhance the effects of exogenous GABA on photosynthetic performance beyond their individual contributions. This is mechanistically grounded in evidence that: (1) light quality regulates photosynthetic electron transport; (2) GABA modulates the C/N balance and antioxidant systems; and (3) these pathways converge at critical regulatory nodes, such as redox homeostasis and chloroplast function. This suggests the potential for synergistic optimization, which is an interaction that has not yet been characterized in CEA photobiology for high value leafy vegetables. This integrated photobiological metabolic approach establishes a novel cultivation framework that optimizes light spectral quality and bioactive supplementation simultaneously. This advances resource efficient CEA systems, enhancing yield, quality, nutritional density and stress resilience to promote sustainable, controlled environment food production.

## Methods

### Plant material and growth conditions

Batavia lettuce seeds (Pakan Bazr Isfahan Co., Isfahan, Iran) were cultivated in 72 sowing trays containing medium-grained perlite (3–5 mm). The culture bed and trays were thoroughly disinfected and washed one day before the start of the experiment, after which the seeds were given a dark treatment for seven days at 25 °C to promote germination. After germination and emergence of the cotyledon leaves, the seedlings were fed half the concentration of Hoagland nutrient solution daily (pH 5.8 ± 0.5; EC 1.5 ± 0.1 µS cm^−1^. After washing the perlite from the roots at the three-leaf stage, the seedlings were transferred to a Nutrient Film Technique (NFT) system (25 °C; relative humidity (RH) 60 ± 2%) and fed the full concentration of the Hoagland nutrient solution (pH 5.8 ± 0.5; EC 2.5 ± 0.1 µS cm^−1^ throughout the growth period.

### Experimental design

The treatments in this experiment included two levels of GABA (Sigma-Aldrich Co., Schnelldorf, Germany): 0 µmol L⁻^1^ (G0), and 25 µmol L⁻^1^ (G25), as well as three light spectra. The GABA was added to the Hoagland nutrient solution used for plant irrigation in the NFT system. Based on previous research, the GABA treatment was applied by adding it to the Hoagland nutrient solution and allowing it to be absorbed by the plants' roots. This allowed the systemic absorption and long-term effects of GABA on the plants to be observed, as well as its effects on the roots, stems and leaves [31, 41]. In order to minimize the effects of transplant shock and ensure that plants reached a physiologically stable vegetative growth stage, GABA application was delayed until 10 days after transplanting. The nutrient solution in the NFT system was continuously replenished throughout the experimental period to compensate for plant uptake. EC and pH were regularly monitored and adjusted to maintain optimal conditions. Following the addition of GABA, the nutrient solution was circulated for 30 min to ensure complete homogeneity, after which the pH was measured and adjusted as necessary using H₂SO₄ or KOH. The pH was subsequently maintained within the target range of 5.8 ± 0.5 throughout the experiment, with daily monitoring to ensure stability. Lighting treatments were provided using different proportions of R and B LED light modules (120 cm × 3 cm; Hangzhou Foison Agricultural Technology Co., Ltd., Hangzhou, China), with peak wavelengths of 450 nm and 660 nm for the R and B LEDs, respectively. The distance between the LEDs and the cultivation surface was 27 cm. The R and B LEDs were chosen as they are recommended for lettuce production [42]. The lighting treatments comprised different combinations of R and B light, including a combination of 70% R and 30% B light (RB:70:30), a combination of 80% R and 20% B light (RB:80:20) and a combination of 90% R and 10% B light (RB:90:10). Each combination had an intensity of 250 ± 20 μmol m⁻^2^ s⁻^1^. Light uniformity was verified daily by measuring the photosynthetic photon flux density (PPFD) at ten randomly selected points across the growing trays using a calibrated quantum sensor (FluorPen). Light intensity variation was kept to within ± 8% (± 20 μmol m⁻^2^ s⁻^1^) at all measurement points. For all lighting treatments in this experiment, the daily light integral (DLI) for growing light (RB) was maintained at a constant level of 14.40 mol m⁻^2^ d⁻^1^ to ensure constant daily light availability for all plants. In the room, no light source except the LEDs were turned on and all the windows were covered with an anti-light coating. Light intensity (PPFD) was measured and verified using a calibrated Sekonic C-7000 spectrometer (Tokyo, Japan), which served as the primary reference instrument for confirming spectral quality and accurately quantifying PPFD across all R:B treatments. Prior to the experiment, the integrated photosynthetic active radiation (PAR) sensor of the FluorPen FP 100-MAX (Photon Systems Instruments, Drásov, Czech Republic) was cross calibrated against the Sekonic C-7000 spectrometer at each specific red to blue (R:B) ratio to ensure consistent measurements. After calibration validation, the FluorPen was used for daily monitoring of light uniformity across the growing trays thanks to its portability and fast measurement capability. The Sekonic spectrometer was used periodically (weekly) to verify spectral stability and recalibrate the FluorPen if needed. This dual instrument approach ensured high precision spectral characterization and practical daily monitoring throughout the experimental period. Two weeks before the start of the experiment, a plant factory system measuring 1 × 2 m and 2.2 m in height was built on four floors (the lower part of the floors was used to house the food solution tank), with each floor measuring 35 cm in height. The system was placed in a 4 × 6 m room in a controlled environment. The room temperature, CO₂ concentration (as determined by a Trotec BZ30 CO₂ air quality data logger from Heinsberg, Germany) and RH were set to 25 °C, 400 ppm and 60%, respectively. Nutrient solution temperature (18 ± 2 °C) and dissolved oxygen (5–6 mg L^−1^) were continuously monitored by an oxygen meter (Lutron DO-5510, Taiwan).

For all treatments, the photoperiod consisted of 18 h of light followed by 6 h of darkness, from 12:00 a.m. to 6:00 a.m., using a model timer (PFTG-28, Pars Fanal, Iran). A FluorPen FP 100-MAX device was used to measure light intensity. Each treatment consisted of 45 plants, and nine replications were used in data collection (200 plants were used in total).

### Sample collection and processing

After 30 days of treatment, nine plants (three in each of three groups) were sampled between 9 and 11 a.m. Their morphology, biochemistry and photosynthetic indices were then determined. Then, nine lettuce plants were randomly selected for further measurements (mixed in triplicate and replicated in three groups). Prior to analysis, the leaves were ground into powder in liquid nitrogen and stored at −70 °C.

### Fast chlorophyll fluorescence transients

To determine the polyphasic chlorophyll fluorescence transients (OJIP test), the FluorPen FP 100-MAX device was used. For this purpose, the fully grown leaves of Batavia lettuce were placed in darkness for 30 min. The chlorophyll fluorescence was then measured nine times at 50 µs intervals: initial stage (F_0_), middle stages (F_j_) at 3 ms, halo phase (F_i_) at 30 ms and final peak stage (F_m_) at 300 ms. Final calculations were performed using PAR-FluorPen software (Strasser et al., 2000). Fluorescence intensity was measured when all PSII reaction centers (RCs) were open. Conversely, F_m_ was derived from the reduction of the primary quinone electron acceptor of PSII (QA) to its oxidized state and represented the maximal chlorophyll fluorescence intensity when all PSII reaction centers were closed. Finally, F_v_/F_m_ represents the maximum photochemical efficiency of PSII, also referred to as the maximum quantum yield of primary photochemistry. Parameters derived from the OJIP test provided information on the energy fluxes of light absorption (ABS), excitation energy trapping (TR_0_) and dissipated energy flux (DI_0_) per reaction center (RC).

### Imaging chlorophyll fluorescence quenching

Samples of mature Batavia lettuce leaves were collected for measuring the slow induction of chlorophyll fluorescence using a FluorCam FC 1000-H (Photon Systems Instruments, Drasov, Czech Republic). These samples were subjected to a 30 min dark adaptation period before immediate utilization. The slow induction kinetics of chlorophyll fluorescence were recorded by applying a series of short, dark adapted excitation flashes. A high intensity saturating pulse (3,900 μmol m⁻^2^ s⁻^1^) was then delivered to transiently inhibit electron flow at the quinone acceptors [43]. This approach enabled the determination of key fluorescence parameters, including minimal fluorescence (F_0_) under dark-adapted conditions and maximal fluorescence (F_m_) during the saturating pulse. Additionally, light adapted maximal fluorescence (F_m’_) was assessed to quantify photochemical quenching capacity [44]. All data processing and kinetic analyses were performed using FluorCam software (v. 7, PSI, Czech Republic) to ensure a high-resolution evaluation of photosynthetic performance.

### Measurement of morphological and yield traits

To determine the morphological data and growth characteristics, such as the fresh weight (FW) and DW of different plant parts (including roots, stems and leaves), nine replications of each treatment were randomly selected every week and weighed accurately. The stems, roots and leaves were separated and placed on white paper. A ladder was positioned next to them as a measurement index. After photographing the plants, Digimizer software (v. 4.1.1.0) was used to determine the number of leaves, LA), root length and stem length. The stem diameter was also measured using a Vernier caliper with an accuracy of 0.02 mm (Mitutoyo, Japan). To determine the DW, the roots, stems and leaves were separated and placed in an oven at 70 °C for 48 h. The plant material was then weighed.

### Relative water content (RWC) of the leaf

One mature leaf was randomly selected from each plant sample, with nine replications in each treatment, in order to measure relative leaf water content. Seven leaf discs were then prepared and weighed to prevent water loss and determine the FW. The discs of each sample were kept separately in a Falcon tube containing 10 ml of distilled water and placed at 4 °C for 24 h. The discs were then weighed until the saturated weight (SW) of each sample was recorded. The discs were then placed in aluminum foil and heated in an oven at 72 °C for 24 h. The weight of the dried leaf discs (DW) was reported in grams per leaf. RWC was calculated using the following formula [45]:

$$\mathrm{RWC}=\frac{\mathrm{FW}-\mathrm{DW}}{\mathrm{SW}-\mathrm{DW}}\times 100$$

### Analysis of photosynthetic pigments

Extraction of photosynthetic pigments (including chlorophyll *a*, *b* and total (*a* + *b*), as well as carotenoids) was conducted using 0.5 g of fresh lettuce leaf samples in 10 mL of 80% acetone until the leaves were fully bleached. Four milliliters of the isolated supernatant were then read in a spectrophotometer (PerkinElmer Lambda 25, Waltham, MA, USA) at wavelengths of 663, 645 and 470 nm [46]. The entire process was conducted under low light conditions to avoid potential interference from light. The results were expressed as mg g⁻^1^ FW.

### Measurement of soluble carbohydrates in the leaves

The total amount of soluble carbohydrates in the leaves was determined using the Irigoyen method (1992). First, 0.1 g of finely ground lettuce leaves in liquid nitrogen was mixed with 13 mL of 80% ethanol. After centrifugation at 5000 rpm for 10 min at 4 °C using a SIGMA 1-14K centrifuge (Darmstadt, Germany), the supernatant solution was separated. Then, 10 mL of 80% ethanol was added to the sediment and the mixture was centrifuged again for 10 min at 5,000 rpm. The above solution was then added to the previous solution, bringing the total volume to 25 mL with 80% ethanol. The samples were placed in a water bath at 100 °C for 15 min and then quickly transferred to cold water. Finally, a spectrophotometer measured the samples at a wavelength of 625 nm. Pure glucose at concentrations of 0, 25, 50, 75 and 100 mg mL^−1^ was used as a standard.

### Quantitative analysis of total anthocyanin content

The total anthocyanin content was quantified using an extraction solution of methanol and hydrochloric acid (99:1, v/v). First, 0.1 g of fresh leaf tissue was pulverized using liquid nitrogen. Then, 10 ml of acidic methanol was added to the leaf tissue. The resulting solution was placed in a dark environment at a temperature of 25 °C for 24 h, after which the sample was centrifuged at 4000 rpm for 5 min. The resulting supernatant was separated and analyzed using a spectrophotometer at a wavelength of 550 nm. Quantification of the anthocyanins was achieved by applying calibration curves derived from malvidin-3-glucoside [47].

### Evaluation of the taste and quality of lettuce

To evaluate the taste and appearance of lettuces, we established a correlation between analytical values and consumer judgement. To taste the lettuce immediately after harvesting, it is washed in distilled water. After drying, the lettuce is cut into small pieces and stored overnight in a plastic container at 4 °C in the refrigerator (to reduce selection errors, a representative sample was randomly selected). The lettuce was then randomly distributed among consumers (men and women aged 18–40) and graded from 1 to 5 based on appearance and taste after consumption (as the score increased from one to five, so did consumer satisfaction) [48].

### Statistical analysis

This experiment was conducted using a completely randomized design (CRD) with two factors: GABA treatment at two levels (G0: control; G25: 25 µmol L⁻^1^ GABA) and R:B light ratio at three levels (70:30, 80:20 and 90:10). This resulted in six treatment combinations, each with nine biological replicates (n = 9). All statistical analyses were performed using SAS software (version 9.2, SAS Institute Inc., Cary, NC, USA). Prior to analysis, data normality and homogeneity of variance were assessed using the Shapiro–Wilk and Levene tests, respectively. As all datasets met these parametric assumptions, a two-way analysis of variance (ANOVA) was conducted to evaluate the main effects and the interactions between the GABA treatment and the light ratio. When significant effects were detected (*p* < 0.05), means were separated using Duncan's multiple range test. Graphical representations were generated using Microsoft Excel (Office 2019, Microsoft Corporation, Redmond, WA, USA).

## Results

### Root to shoot ratio of Batavia lettuce

**Fig. 1:**
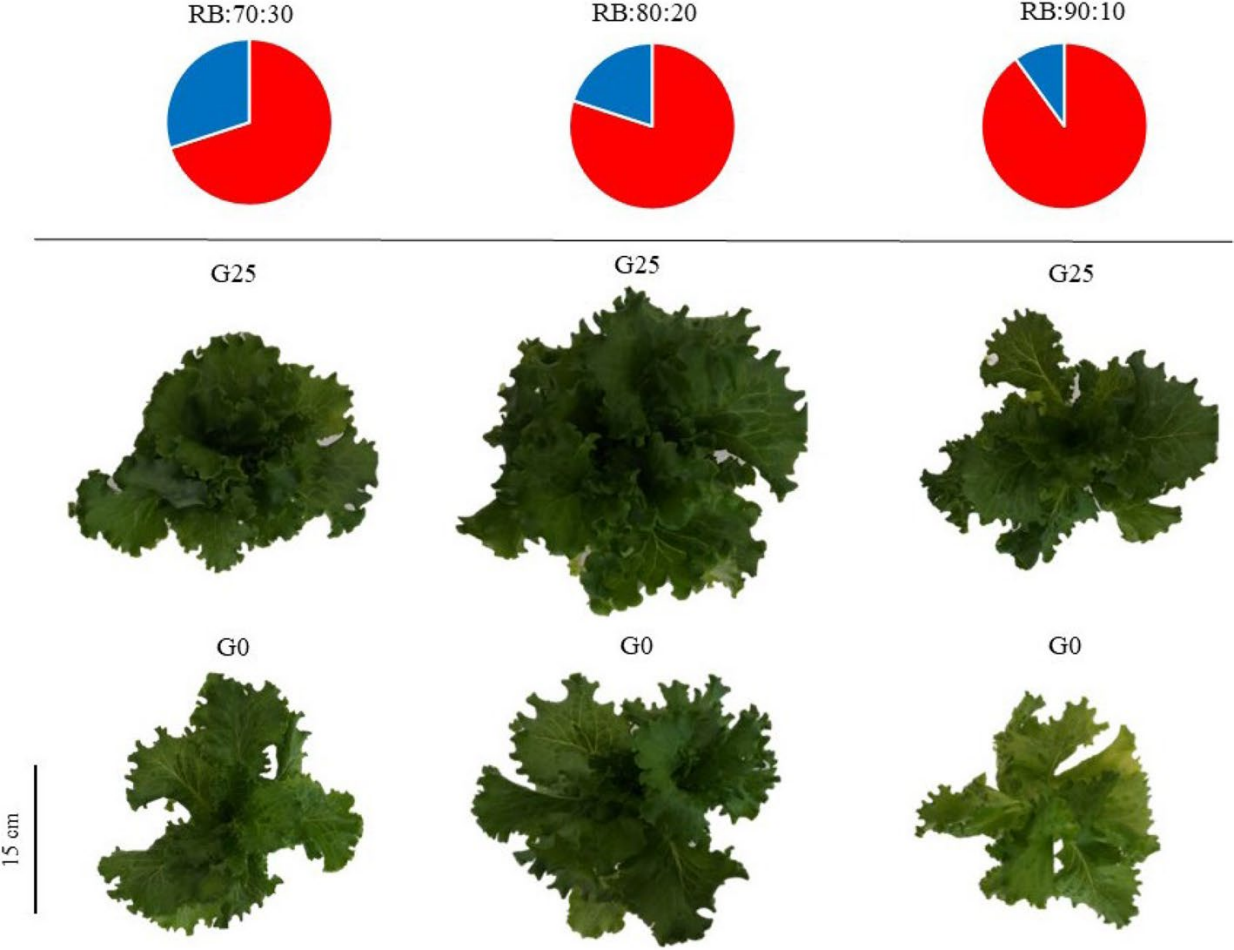
Morphology of Batavia lettuce plants after 30 days. Different ratios of the red (R) and blue (B) light spectrum were used: RB:70:30, RB:80:20 and RB:90:10. The plants were grown for 30 days under different red:blue light ratios (70:30, 80:20 and 90:10) at 250 ± 20 μmol m⁻^2^s⁻^1^ PPFD. The plants were irrigated with a Hoagland nutrient solution containing 25 μmol L⁻.^1^ of γ-aminobutyric acid (GABA) (G25) or no GABA (G0)

Plants respond to different environmental conditions by inducing metabolic changes in their aerial organs and transferring carbohydrates to various parts of the plant. These metabolic adjustments often involve the synthesis, mobilization and redistribution of carbon, which is then transported to different parts of the plant, including the roots, developing leaves and stems, in order to support growth and the plants response to environmental changes and adaptation [49] (Fig. 1).

**Fig. 2:**
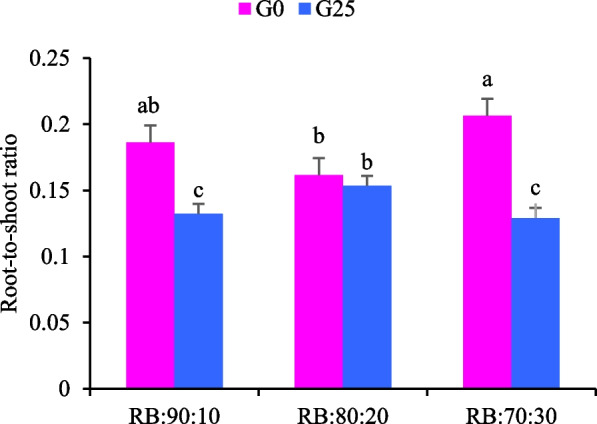
Dry weight (DW) of the root-to-shoot ratio (stem and leaf) in Batavia lettuce plants. Different ratios of the red (R) and blue (B) light spectrum were used: RB:70:30, RB:80:20 and RB:90:10. The plants were grown for 30 days under different red:blue light ratios (70:30, 80:20 and 90:10) at 250 ± 20 μmol m⁻^2^s⁻^1^ PPFD. The plants were irrigated with a Hoagland nutrient solution containing 25 μmol L⁻^1^ of γ-aminobutyric acid (GABA) (G25) or without GABA (G0). Data represents the mean ± SE of nine replicates per treatment. Different letters indicate statistically significant differences at *p* < 0.05 according to Duncan's multiple range test

The root to shoot ratio of Batavia lettuce was significantly affected by both GABA application and light spectrum treatments (*p* ≤ 0.05). Plants treated with G25 generally exhibited lower root-to-shoot ratios compared to control plants (G0) across most light treatments, indicating greater biomass allocation to shoot tissues (Fig. 2). With an increase in the amount of B light in the LED light spectrum composition (RB:70:30), plant biomass increased towards the root, thereby increasing the root to shoot ratio in lettuce grown under this treatment. The RB:90:10 + G25 treatment exhibited the lowest root to shoot ratio, with a 44% reduction compared to the RB:70:30 + G0 control. Plants under the RB:80:20 light spectrum with the G25 treatment and without GABA application (G0) did not show significant differences in the root to shoot ratio, unlike the other two light spectra (Fig. 2). Overall, this study showed that the interaction between GABA and increasing the R to B light spectrum ratio caused the transfer of maximum biomass to the aerial organs (Fig. 2).

### Quantitative analysis of lettuce growth over four consecutive weeks

**Fig. 3:**
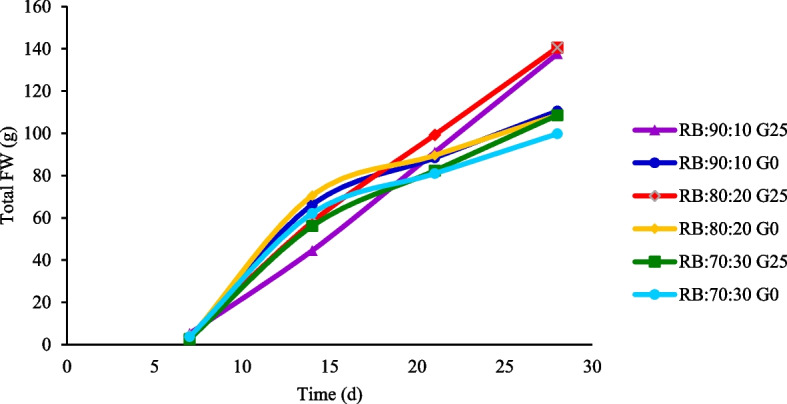
Total fresh weight (the FW of root, leaf, and stem) of Batavia lettuce during four consecutive weeks. Different ratios of red (R) and blue (B) light spectrum (RB:70:30, RB:80:20, and RB:90:10). Plants were grown for 30 days under different red (R):blue (B) light ratios (70:30, 80:20, 90:10) at 250 ± 20 μmol m⁻^2^s⁻^1^ PPFD. Plants were irrigated with Hoagland nutrient solution containing 25 μmol L⁻^1^ γ-aminobutyric acid (GABA) (G25), and without GABA (G0). Data represent means ± SE of nine replicates per treatment. Different letters indicate statistically significant differences at *p* < 0.05 according to Duncan's multiple range test

When studying and measuring the trend of changes in lettuce FW during the 30 days treatment period after transferring the seedlings to the plant factory structure, the RB:80:20 + G0 treatment exhibited the greatest growth, as measured by root, leaf and stem FW. There was no statistically significant difference between this treatment and the RB:90:10 + G0 treatment (*p* > 0.05). On days 14 and 21, applying GABA (G25) and increasing the amount of B light in LEDs significantly increased total plant FW under the RB:80:20 and RB:90:10 light spectra. On day 28, the highest growth was observed in the RB:80:20 + G25 treatment (140.55 g), which was marginally higher than in the RB:90:10 + G25 treatment (137.57 g) (Fig. 3).

### Interactive effects of GABA and red:blue light ratios on lettuce root growth

**Fig. 4:**
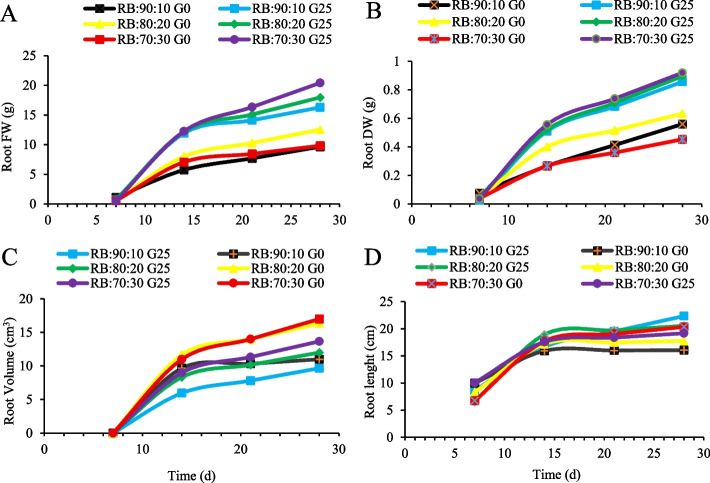
Root fresh weight (FW) (**A**), root dry weight (DW) (**B**), root volume (**C**), and root length (**D**) of Batavia lettuce during four consecutive weeks. Different ratios of red (R), and blue (B) light spectrum (RB:70:30, RB:80:20, and RB:90:10). Plants were grown for 30 days under different red (R):blue (B) light ratios (70:30, 80:20, 90:10) at 250 ± 20 μmol m⁻^2^s⁻^1^ PPFD. Plants were irrigated with Hoagland nutrient solution containing 25 μmol L⁻.^1^ γ-aminobutyric acid (GABA) (G25) and without GABA (G0)

Examining changes in root growth over four consecutive weeks revealed that, on day 7 of lettuce seedling growth and planting in the plant factory, the highest FW and DW of the root were observed in the RB:90:10 + G0 treatment. However, subsequent weeks saw an increase in FW and DW of the root in all light treatments following GABA (G25) application. Conversely, the RB:70:30 + G25 treatment recorded the highest root DW on day 28, while the same light spectrum (RB:70:30) with no GABA application (G0) recorded the lowest root DW (Fig. 4A and B). Measuring the volume of lettuce roots over four consecutive weeks revealed that root volume was initially low and remained nearly constant across all treatments. The highest root volume was related to the RB:80:20 + G0 treatment on the 14th day. From day 21 to day 28, the highest root volume was observed in the treatment without GABA application (G0) under the RB:70:30 and RB:80:20 light spectra. This increase in root volume was attributed to the higher B:R light ratio (Fig. 4C). On day 28, the lowest root volume was recorded in the RB:90:10 + G25 treatment (Fig. 4C).

The longest root length was observed on day 7 for the RB:70:30 + G0 treatment, which was not significantly different to the RB:90:10 + G0 treatment. GABA application (G25) and the RB:80:20 light spectrum promoted increased root length on days 14 and 21. Contrary to the adverse effect of GABA on root volume increase (Fig. 4C), the data showed that its application (G25) positively impacted root length increase (Fig. 8D). The highest root length was achieved with the G25 treatment (RB:80:20 + G25 and RB:90:10 + G25) in the final week. Furthermore, as the R:B light ratio increased, so did root longitudinal growth, with the RB:90:10 + G25 treatment achieving the greatest root length by day 28 (Fig. 4D).

### Leaf area and leaf numbers of Batavia lettuce

**Fig. 5:**
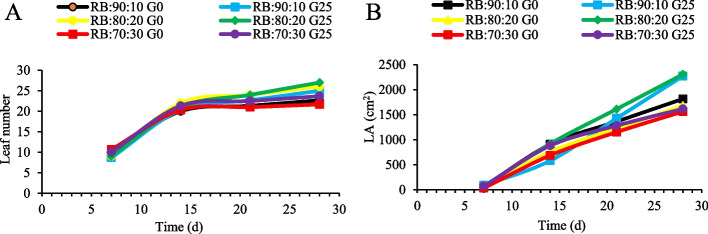
Leaf number (**A**) and leaf area (LA) (**B**) of Batavia lettuce over four consecutive weeks. Different ratios of the red (R) and blue (B) light spectrum were used: RB:70:30, RB:80:20 and RB:90:10. The plants were fertilized with a Hoagland solution containing 25 μmol L^−1^ GABA (G25) and no GABA (G0), for 30 days. The photosynthetic photon flux density was adjusted to 250 ± 20 μmol m⁻^2^ s⁻^1^ during the experiment. Nine replicates were examined per treatment

When the number of lettuce leaves and LA were measured over four consecutive weeks, no significant differences (*p* < 0.05) were observed between the plants under different treatments during the first week (7 days) after application. However, over the following weeks, the application of GABA increased and the number of leaves. On day 21, the RB:70:30 + G25 treatment had the highest number of leaves. On day 28, an increase in the number of leaves was observed in the RB:80:20 + G25 treatment (Fig. 5A). The amount of LA increased in the G25 treatment in the RB:90:10 and RB:80:20 spectra on days 21 and 28. Application of GABA (G25) and an increase in the R spectrum ratio increased LA development (Fig. 5B).

### Influence of GABA and R:B light levels on leaf photosynthetic pigment content

**Fig. 6:**
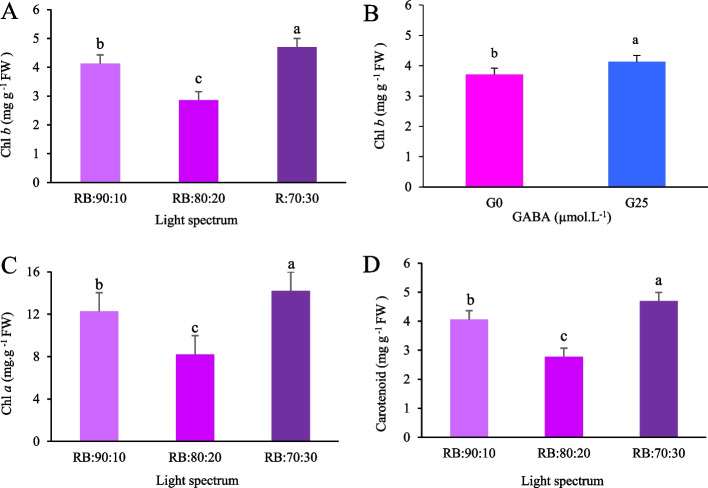
Effect of light spectrum (**A**) and GABA (**B**) on chlorophyll *b* (Chl *b*), effect of light spectrum on chlorophyll *a* (Chl *a*) (**C**) and effect of light spectrum on carotenoid content (**D**) in Batavia lettuce plants under different ratios of red (R) and blue (B) light spectrum (RB:70:30, RB:80:20, and RB:90:10). Plants were grown for 30 days under different red (R):blue (B) light ratios (70:30, 80:20, 90:10) at 250 ± 20 μmol m⁻^2^s⁻^1^ PPFD. Plants were irrigated with Hoagland nutrient solution containing 25 μmol L⁻^1^ γ-aminobutyric acid (GABA) (G25), and without GABA (G0). Data represent means ± SE of nine replicates per treatment. Different letters indicate statistically significant differences at *p* < 0.05 according to Duncan's multiple range test

Analysis of photosynthetic pigments revealed significant variations in the content of Chl *a* and Chl *b* among the different treatments. Both the light spectrum and GABA application showed significant main effects on Chl *b* content. The RB:70:30 treatment was associated with the highest Chl *b* content, while the RB:80:20 treatment was associated with the lowest (Fig. 6A). The RB:30:70 spectrum increased Chl *b* content by 1.67 times compared to the RB:80:20 spectrum (Fig. 6A). In terms of the main effect of GABA levels, the highest Chl *b* content was associated with the G25 treatment, averaging 4.13 mg g⁻^1^ FW (Fig. 6B). Chl *a* content was affected by the light spectrum (*p* < 0.01) and consistent with the Chl *b* results, was highest in the RB:70:30 treatment and lowest in the RB:80:20 treatment (Fig. 6C). Lettuce grown under the RB:70:30 spectrum showed 1.73 times increase in Chl *a* compared to the RB:80:20 spectrum (Fig. 6C). Carotenoid accumulation in the leaves was significantly influenced by the light spectrum (*p* < 0.01) but remained unaffected by GABA application (*p* > 0.05). Similar to the patterns observed for chlorophyll, the amount of carotenoids increased under the RB:70:30 light spectrum, while the lowest amount was recorded under the RB:80:20 spectrum (Fig. 6D). Lettuce grown under the RB:70:30 treatment demonstrated 1.69 times increase in carotenoid content compared to the RB:80:20 treatment (Fig. 6D).

### Relative Water Content (RWC) of leaves under different red:blue light spectra

**Fig. 7:**
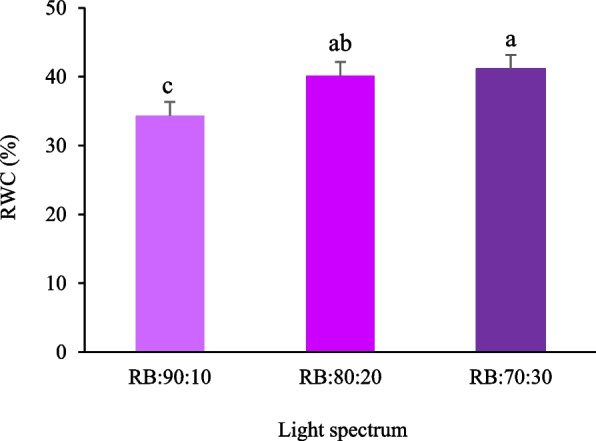
Relative water content (RWC) in Batavia lettuce leaves under different ratios of red (R) and blue (B) light spectrum (RB:70:30, RB:80:20, and RB:90:10), under fixed light intensity of 250 ± 10 µmol.m^−2^s^−1^. Plants were grown for 30 days under different red (R):blue (B) light ratios (70:30, 80:20, 90:10) at 250 ± 20 μmol m⁻^2^s⁻^1^ PPFD. Plants were irrigated with Hoagland nutrient solution containing 25 μmol L⁻^1^ γ-aminobutyric acid (GABA) (G25), and without GABA (G0). Data represent means ± SE of nine replicates per treatment. Different letters indicate statistically significant differences at *p* < 0.05 according to Duncan's multiple range test

Analysis of variance revealed that the light spectrum had a significant effect on leaf RWC at *p* ≤ 0.05. The results showed that RWC remained significantly higher in the RB:70:30 treatment than in the RB:80:20 treatment (*p* ≤ 0.05). The lowest RWC value was observed in the RB:90:10 treatment (Fig. 7). Plants grown under the RB:70:30 treatment had an RWC value that was 1.91 times higher than those grown under the RB:90:10 treatment (Fig. 7).

### Photosynthetic performance of Batavia lettuce

**Fig. 8:**
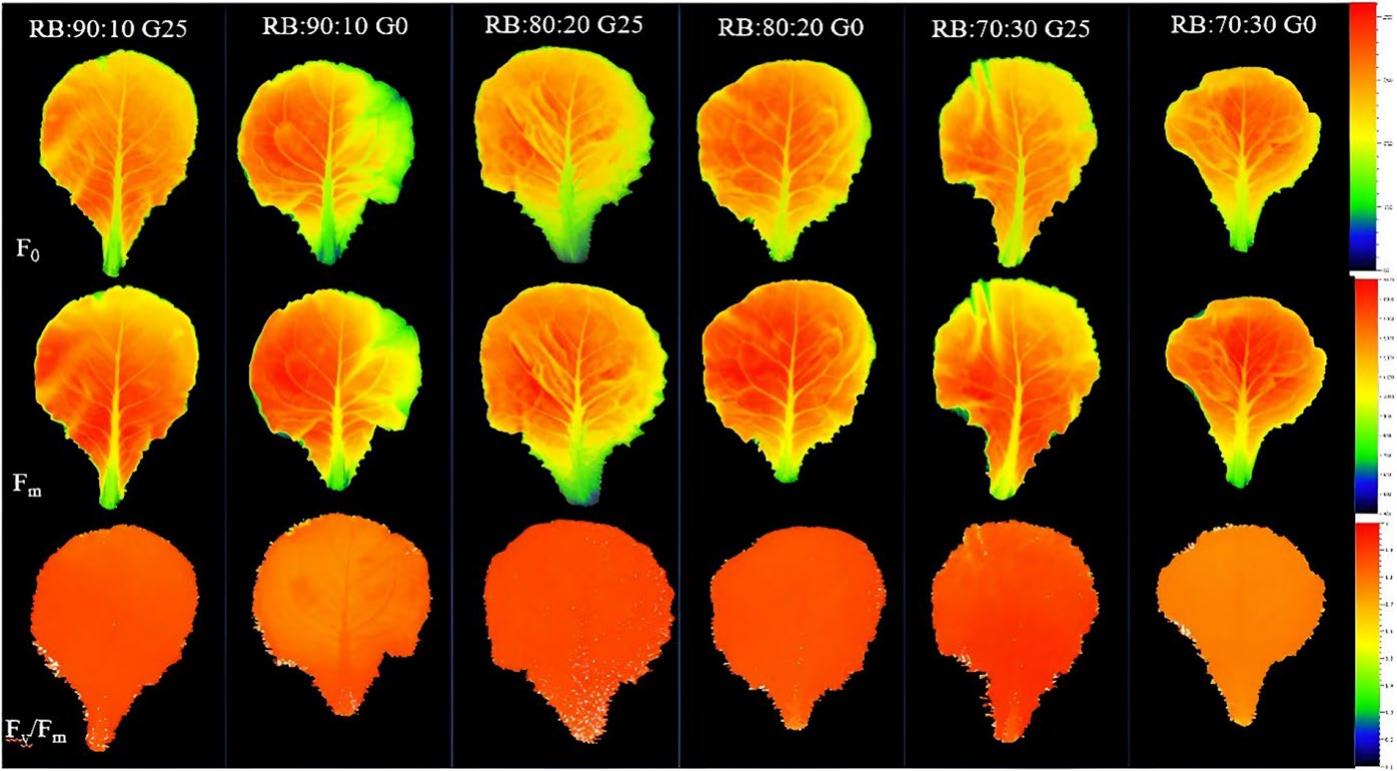
shows pseudo-color images of the maximum quantum yield of PSII (F_v_/F_m_), F_0_ and F_m_ from leaves sampled from Batavia lettuce that had been exposed for 30 days to different treatments. The light spectrum was at different ratios of red (R) and blue (B) (70:30, 80:20 and 90:10) with a PPFD of 250 ± 20 μmol m⁻^2^ s⁻^1^. The plants were fertilized with a Hoagland solution containing 25 μmol L.^−1^ γ-aminobutyric acid (GABA) (G25) or no GABA (G0)

The interactive effects of light quality and GABA supplementation on the photosynthetic performance of Batavia lettuce leaves were evaluated using chlorophyll fluorescence imaging and OJIP transient analysis. The spatial distribution patterns of the fluorescence parameters were visualized using pseudo color images representing F_0_, F_m_ and the PSII quantum yield (F_v_/F_m_). Plants exposed to the RB:80:20 treatment exhibited the highest F_v_/F_m_ values, as indicated by warmer coloration (red–orange) in the pseudo-color fluorescence images (Fig. 8), suggesting optimal PSII functionality. The color intensity scale (right panel, Fig. 8) shows that higher F_v_/F_m_ values corresponded to warmer colors, while lower values were represented by cooler colors (blue-green). GABA supplementation (G25) significantly enhanced PSII photochemical efficiency compared to control plants. Plants treated with GABA (G25) consistently exhibited higher F_v_/F_m_ values across all light treatments, suggesting enhanced photosynthetic capacity.

### Polyphasic chlorophyll fluorescence transient (OJIP)

**Fig. 9:**
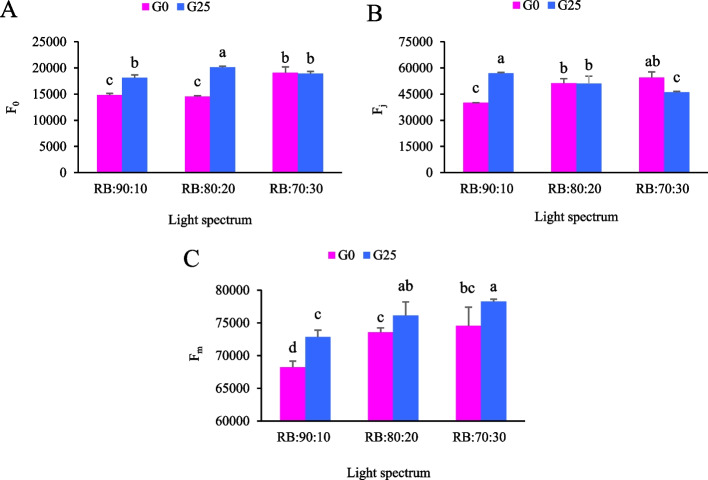
Polyphasic chlorophyll fluorescence parameters in Batavia lettuce leaves under different red:blue light ratios and GABA treatments. **A** Minimum fluorescence when all PSII reaction centers (RCs) are open (O-step) (F_o_), **B** Fluorescence intensity at the J-step (F_j_), and **C** Maximum fluorescence, when all PSII RCs are closed (F_m_). Plants were grown for 30 days under different red (R):blue (B) light ratios (70:30, 80:20, 90:10) at 250 ± 20 μmol m⁻^2^s⁻^1^ PPFD. Plants were irrigated with Hoagland nutrient solution containing 25 μmol. L⁻^1^ γ-aminobutyric acid (GABA) (G25), and without GABA (G0). Data represent means ± SE of nine replicates per treatment. Different letters indicate statistically significant differences at *p* < 0.05 according to Duncan's multiple range test

Polyphasic chlorophyll fluorescence transients (the OJIP test) were measured in the fully expanded leaves of Batavia lettuce plants that were subjected to different ratios of R:B light and different concentrations of GABA. F_0_ values varied significantly between treatments. The lowest F_0_ value was recorded in plants exposed to the RB: 80:20 + G0 treatment. GABA increased this parameter's value, with the maximum F_0_ fluorescence observed in the photosynthetic apparatus of lettuce under the RB:80:20 + G25 treatment (Fig. 9A). The highest F_j_ value was observed in lettuce treated with RB:90:10 + G25; however, there was no statistically significant change in plants treated with RB:30:70 and G0 (Fig. 9B). The lowest F_j_ value was also recorded in lettuce exposed to RB:90:10 + G0 (Fig. 9B). The F_j_ value in the RB:90:10 + G25 treatment was 1.4 times higher than in the RB:90:10 + G0 treatment (Fig. 9B). The maximum F_m_ value was obtained in the RB 70:30 + G25 treatment, which was statistically similar to the RB:80:20 + G25 treatment, but significantly different to all the other treatments. The lowest F_m_ was observed in the RB:90:10 + G0 treatment (Fig. 9C). F_m_ results showed 1.147 times increase in lettuce growth in the RB:70:30 + G25 treatment compared to the RB:90:10 + G0 treatment (Fig. 9C).

**Fig. 10:**
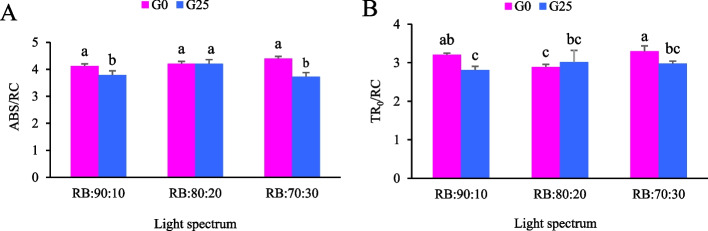
**A** The specific energy fluxes per RC for energy absorption (ABS/RC) and **B** Trapped energy flux (TR_0_/RC) in Batavia lettuce leaves under different treatments. Different ratios of red (R) and blue (B) light spectrum (RB:70:30, RB:80:20, and RB:90:10). Plants were grown for 30 days under different red (R):blue (B) light ratios (70:30, 80:20, 90:10) at 250 ± 20 μmol m⁻^2^s⁻^1^ PPFD. Plants were irrigated with Hoagland nutrient solution containing 25 μmol. L⁻^1^ γ-aminobutyric acid (GABA) (G25), and without GABA (G0). Data represent means ± SE of nine replicates per treatment. Different letters indicate statistically significant differences at *p* < 0.05 according to Duncan's multiple range test

The energy flux parameters (ABS/RC and TR_0_/RC) were measured in mature leaves after 30 days under different R:B light spectrum levels and GABA treatments (Fig. 10A, and B). The TR_0_/RC results, indicating trapped energy flux at each RC, and the ABS/RC results, indicating the specific energy flux per RC for energy absorption, showed the highest levels in plants exposed to a light spectrum with a higher percentage of B light and no GABA treatment (RB:70:30 + G0). These levels were not significantly different to those observed in the RB:90:10 + G0 treatment (Fig. 10, A-B).

**Fig. 11:**
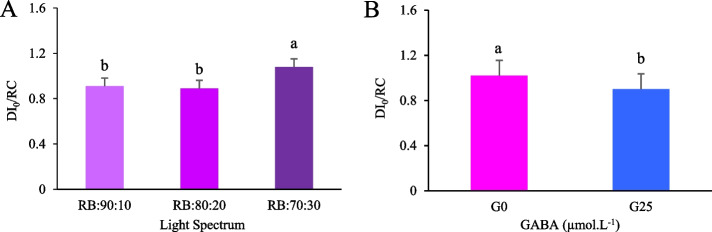
Dissipated energy flux (DI_0_/RC) in Batavia lettuce leaves under different ratios of red (R) and blue (B) light spectrum (RB:70:30, RB:80:20 and RB:90:10) (**A**) and plants were fertilized for 30 days under 250 ± 20 μmol m⁻^2^s⁻^1^ PPFD with Hoagland solution containing 25 μmol. L⁻^1^ γ-aminobutyric acid (GABA) (G25), and without GABA (G0) (**B**). Data represent means ± SE of nine replicates per treatment. Different letters indicate statistically significant differences at *p* < 0.05 according to Duncan's multiple range test

For the DI_0_/RC parameter, both the light spectrum and the application of GABA showed significant main effects (*p* ≤ 0.05), but their interaction was not statistically significantThe highest DI_0_/RC values among the light spectrum treatments were the RB:70:30 ratio, which showed 1.34 times increase compared to the RB:90:10 treatment (Fig. 11A). The lowest DI_0_/RC values were recorded under the RB:80:20 spectrum and did not differ significantly from those under the RB:90:10 spectrum. Regarding GABA application, the G25 treatment resulted in the highest DI_0_/RC value (1.029), showing a significant increase compared to the control (G0) treatment (Fig. 11B).

### Soluble carbohydrates in Batavia leaves

**Fig. 12:**
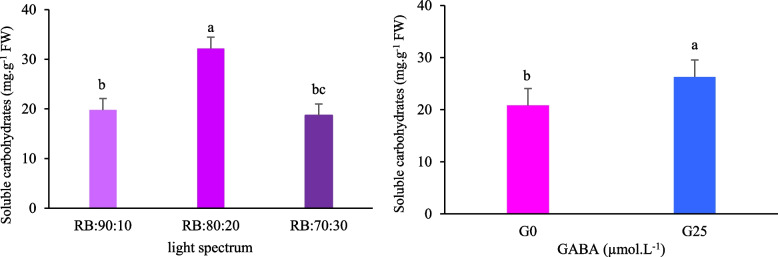
Soluble carbohydrate content in Batavia lettuce leaves under **A** different ratios of red (R), and blue (B) light spectrum (RB:70:30, RB:80:20, and RB:90:10), under fixed light intensity of 250 ± 10 µmol.m^−2^s^−1^. **B** Plants were grown for 30 days under different red (R):blue (B) light ratios (70:30, 80:20, 90:10) at 250 ± 20 μmol m⁻^2^s⁻^1^ PPFD. Plants were irrigated with Hoagland nutrient solution containing 25 μmol L⁻^1^ γ-aminobutyric acid (GABA) (G25) and without GABA (G0). Data represent means ± SE of nine replicates per treatment. Different letters indicate statistically significant differences at *p* < 0.05 according to Duncan's multiple range test

Data analysis revealed that GABA and light spectrum treatments had a significant impact on soluble carbohydrate content in leaves when administered separately (Fig. 12, A-B). Soluble carbohydrates accumulated primarily in plants exposed to the RB:80:20 treatment. The lowest concentration of soluble carbohydrates was observed in the RB:70:30 treatment and was not significantly different to the RB:90:10 treatment (Fig. 12A). GABA application resulted in an increase in soluble carbohydrates of 1.26 times compared to the treatment without GABA application (Fig. 12B).

### Enhanced anthocyanin content with GABA and high blue: red light ratio

**Fig. 13:**
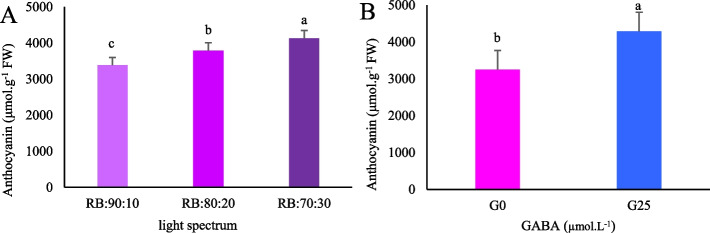
Total anthocyanins content in Batavia lettuce leaves under **A** different ratios of red (R), and blue (B) light spectrum (RB:70:30, RB:80:20, and RB:90:10), under fixed light intensity of 250 ± 10 µmol.m^−2^s^−1^. **B** Plants were grown for 30 days under different red (R):blue (B) light ratios (70:30, 80:20, 90:10) at 250 ± 20 μmol m⁻^2^s⁻^1^ PPFD. Plants were irrigated with Hoagland nutrient solution containing 25 μmol L⁻^1^ γ-aminobutyric acid (GABA) (G25), and without GABA (G0). Data represent means ± SE of nine replicates per treatment. Different letters indicate statistically significant differences at *p* < 0.05 according to Duncan's multiple range test

The amount of anthocyanin in the leaf was affected by the main effects of both GABA and the light spectrum (*p* < 0.01). Lettuce plants grown under an RB:70:30 light treatment exhibited the highest total anthocyanin content, whereas those grown under an RB:90:10 light treatment exhibited the lowest. The anthocyanin content of the RB:70:30 treatment was 1.22 times higher than that extracted from the leaves of plants treated with RB:90:10 (Fig. 13A). Overall, the anthocyanin content of Batavia lettuce leaves increased significantly under GABA supplementation (G25) compared to the GABA control treatment (G0) (Fig. 13B).

### Quality indicators of the leaves of Batavia lettuce

**Fig. 14:**
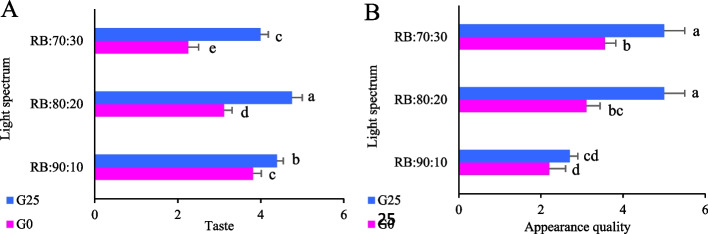
Appearance quality of fresh lettuce (**A**) and taste of lettuce (**B**). Different ratios of red (R) and blue (B) light spectrum (RB:70:30, RB:80:20, and RB:90:10). Plants were grown for 30 days under different red (R):blue (B) light ratios (70:30, 80:20, 90:10) at 250 ± 20 μmol m⁻^2^s⁻^1^ PPFD. Plants were irrigated with Hoagland nutrient solution containing 25 μmol L⁻^1^ γ-aminobutyric acid (GABA) (G25), and without GABA (G0). Data represent means ± SE of nine replicates per treatment. Different letters indicate statistically significant differences at *p* < 0.05 according to Duncan's multiple range test

A consumer evaluation of lettuce quality indices, conducted using leaves from the outer part of each lettuce head, revealed that lettuces grown under the RB:80:20 and RB:70:30 light spectra in the G25 treatment received the highest appearance quality scores, with an average maximum score of 5. These scores were significantly higher than those received by lettuces grown under other treatments (*p* < 0.05). The lowest appearance quality was observed in lettuce grown under the RB:90:10 spectrum in the G25 and G0 treatments (Fig. 14A). In terms of taste, the RB:80:20 + G25 treatment received the highest consumer score. In contrast, more than 50% of panelists rated the taste of lettuces grown under the RB:70:30 + G0 treatment as worse than those grown under other treatments (Fig. 14B).

## Discussion

### Blue light induced carbon partitioning to the root and GABA increased shoot biomass

Light quality is a key factor determining plant growth and biomass allocation through wavelength specific activation of photoreceptors and downstream signaling pathways [32]. In this study, plants grown under the RB:80:20 spectrum showed maximum LA and leaf FW, indicating that R enriched light optimizes photosynthetic performance and canopy development. In contrast, a higher proportion of B light (RB:70:30 + G0) suppressed shoot growth, an effect that was largely attenuated by GABA supplementation (RB:70:30 + G25; Fig. 1). Exogenous GABA has been reported to improve photosynthesis, reduce ROS, and activate antioxidant enzymes in several crops, including *Oryza sativa* and *Vigna radiata* [50, 51]. In *Lactuca sativa*, GABA supplementation likely increased leaf expansion, maintained chloroplast integrity, and directed resources toward stem growth rather than defense [31].

B light limits vegetative growth in *Arabidopsis thaliana* by activating cryptochrome and phytochrome interacting factor (PIF) signaling, which reduces elongation and alters auxin responses [52]. In addition, short wavelength, high energy photons increase ROS production and activate antioxidant defense systems, diverting absorbed carbon from growth to photoprotection and defense metabolism [39]. This trade off likely explains the reduction in shoot biomass observed under RB:70:30 conditions. Conversely, spectra with a higher proportion of R light (RB:80:20 and RB:90:10) increase LA and shoot biomass by increasing photosynthesis and photomorphogenic responses, while root growth is not proportionally increased [13, 53].

B enriched light also increased the root to shoot ratio (Fig. 6), indicating its stimulatory effect on root development. Physiogically, B light increases auxin biosynthesis and transport, in part through upregulation of YUCCA genes, thereby increasing lateral root initiation and elongation. This preferential allocation to the root under high B light contrasts with the high level of R spectra that promote shoot expansion. In a study on *Brassica rapa* var. chinensis, higher R light increased shoot biomass while higher B light increased root development [53]. Root applied GABA consistently decreased the root to shoot ratio across light treatments (Fig. 2), indicating a shift in biomass allocation toward shoots that is beneficial in leafy crops [38]. This response likely involves root to shoot signaling, as GABA acts as a mobile metabolite capable of long distance transport through the vascular system [32]. GABA modulates nitrogen uptake and assimilation, carbon–nitrogen balance, and phytohormone signaling, particularly auxin and cytokinin, which together enhance shoot storage capacity [54]. In addition, GABA may indirectly affect shoot growth by altering the metabolic and redox status of the root and by influencing xylem transmitted signals such as amino acids, organic acids, or hormones, thereby supporting leaf expansion and biomass accumulation even under B light enriched conditions (RB:70:30 + G25).

Mechanistically, the observed increase in LA under GABA treatment was primarily due to cell expansion rather than increased cell proliferation. GABA increases the efficiency of photosynthesis, particularly PSII light utilization, and maintains redox balance [55], thereby increasing the availability of carbohydrates for cell wall relaxation and turgor induced enlargement. R enriched spectra (RB:80:20) also activated phytochrome signaling and increased the expression of genes associated with cell expansion, such as EXPANSINS, rather than stimulating mitotic activity [56]. The lack of a proportional increase in leaf number under GABA treatment further supports cell expansion as the dominant mechanism underlying the increase in LA.

### Spectral regulation of biomass partitioning and GABA mediated growth regulation

Monitoring changes in plant growth parameters at uniform intervals allows precise analysis of developmental trends and treatment effects [57]. Over the 30 days experimental period, total FW exhibited spectrum dependent flexibility and GABA responsiveness. Under the RB:80:20 light spectrum with a high R component, total FW was maximized, likely due to enhanced photosynthetic efficiency, as evidenced by elevated photosynthetic activity (Fig. 7). By the fourth week, GABA supplementation (G25) further increased FW (Fig. 3) and redirected carbon allocation toward leaves (Fig. 9B).

Conversely, a higher B light fraction (RB:70:30 + G0) reduced LA by 25% and decreased leaf number (Fig. 5), consistent with impaired PSII efficiency in *Cucumis sativus* and *Brassica chinensis* under high B light [58, 59]. These effects likely result from photoinhibition and ROS accumulation, both mitigated by GABA through antioxidant enzyme activation and redox homeostasis maintenance [60]. GABA application under RB:80:20 + G25 also enhanced LA by balancing PSI/PSII excitation and alleviating “Red Light Syndrome”. Increased LA consequently improves photosynthetic capacity through a larger light absorbing surface and optimal R:B ratio [61]. Additionally, GABA modulates stomatal conductance and chlorophyll biosynthesis, supporting sustained leaf expansion and improved light absorption [2, 62].

Overall, these data indicate a trade off in growth strategy: R light promotes structural (leaf) investment, whereas GABA refines biomass allocation by maintaining redox stability and enhancing nitrogen uptake [63]. In *Solanum lycopersicum* seedlings, flexible responses to spectral shifts were linked to regulation of GABA transaminase gene (GABA-T1) expression [30]. High R spectra (RB:80:20 and RB:90:10) activated phytochrome signaling, upregulating EXPANSIN and LHCB genes that promote leaf expansion and a more open canopy architecture [56]. Root morphology was also significantly influenced by both spectrum and GABA (Fig. 4). Elevated B light (RB:70:30 + G0) inhibited internode elongation and triggered cryptochrome mediated suppression of auxin efflux, producing compact plants with increased root proliferation [56, 64]. In contrast, GABA treatment (RB:80:20 + G25) promoted root elongation without volumetric expansion, implying modulation of aluminum activated malate transporter (ALMT) fluxes and ROS inhibition [65].However, RB:90:10 + G25 reduced root volume, likely due to auxin transporter inhibition and limited carbon allocation to roots [56].

Integration of GABA into TCA cycle and its interaction with phytohormones such as auxin and cytokinin support leaf expansion while maintaining metabolic equilibrium [32].Collectively, the synergistic action of optimal R:B light spectra and GABA supplementation demonstrates strong potential for improving canopy architecture, photosynthetic efficiency, and resource-use optimization in CEA systems [66].

### Blue light and GABA supplementation synergistically enhance carbon fixation and chlorophyll biosynthesis

The spectral composition of light plays a critical role in regulating plant growth by modulating pigment biosynthesis, photosynthetic efficiency and photoprotective mechanisms. In this study, the RB:70:30 spectrum, which is characterized by a higher proportion of B light, was found to significantly increase the concentrations of Chl *a*, Chl *b* and carotenoids. This response is consistent with the well documented 'blue light syndrome, whereby elevated B light enhances pigment synthesis while simultaneously restricting leaf expansion, resulting in a compact morphology and reduced LA. The higher B light fraction likely enhanced PSII activity by improving electron transport efficiency and optimizing the NADPH/ATP ratio for downstream photochemical reactions. Additionally, B light increased stomatal conductance and CO₂ diffusion capacity, thereby supporting photosynthetic carbon assimilation despite higher photochemical energy demands. However, intensified pigment biosynthesis under high B light conditions diverts metabolic resources towards photoprotection and antioxidant defense pathways, ultimately limiting leaf expansion and biomass accumulation [67].

By contrast, the RB:80:20 spectrum, which is enriched in R light, promoted greater LA and shoot biomass accumulation by stimulating photosynthetic carbon fixation. GABA supplementation under this spectral treatment further enhanced leaf expansion and optimized carbon partitioning to shoot tissues, potentially mitigating the adverse effects of R light dominance on stomatal regulation and nutrient acquisition. GABA application also stabilized pigment concentrations, particularly Chl *b*, by protecting thylakoid membranes from ROS induced oxidative damage and enhancing the structural stability of photosystems. These findings demonstrate that, although elevated B light increases chlorophyll content, this does not necessarily lead to increased biomass production. Ultimately, the metabolic balance between photoprotection and growth related carbon allocation determines plant productivity [68].

The integration of GABA with the light spectrum provided a synergistic effect on carbon fixation and carbohydrate accumulation. R light, while promoting photosynthesis, can lead to oxidative stress and water loss if not balanced by B light. In this study, GABA enhanced photosynthetic efficiency and carbohydrate transport, optimizing energy distribution and improving plant stress tolerance. Moreover, GABA played a critical role in regulating carbon–nitrogen metabolism, further supporting efficient growth and biomass accumulation [63].

Overall, this study demonstrates that a balanced light spectrum, augmented by GABA supplementation, optimizes photosynthesis, promotes chlorophyll biosynthesis, and enhances plant productivity and stress resilience in controlled environments [11, 30, 63]. Taken together, these results highlight the importance of evaluating both pigment content and photosynthetic efficiency to accurately assess plant growth under varying light spectra.

### Red light, by closing the stomata, limits water absorption in non-stressed conditions

Light exposure significantly influences plant water relations by integrating photoreceptor signaling with stomatal function, hydraulic conductivity, and osmotic regulation. B light activates phototropin receptors in guard cells, triggering stomatal opening through H⁺ ATPase-mediated hyperpolarization and potassium influx. This leads to increased turgor in guard cells, wider stomatal apertures, and enhanced CO₂ diffusion and transpiration, promoting water uptake from roots to balance the transpiration driven mass flow. The RB:70:30 light spectrum, with a balanced B light proportion, maintains RWC by ensuring effective stomatal activation without excessive transpiration loss, improving water use efficiency and carbon assimilation [69].

The positive impact of B light on RWC has been observed in species like *Fragaria* × *ananassa* and *Vicia faba* [70, 71], where B light enhanced stomatal responsiveness and delayed wilting. The RB:70:30 treatment likely improves root hydraulic conductivity and aquaporin (PIP) activity, facilitating water uptake and osmotic regulation. This coordination between stomatal function and water uptake ensures adequate leaf hydration, supports cell expansion, and facilitates efficient photosynthesis and enzyme functionality, especially under fluctuating evaporative demand.

In contrast, R light dominance tends to cause partial stomatal closure due to weaker activation of B light sensitive phototropins, favoring water conservation over gas exchange. This leads to reduced transpiration and CO₂ assimilation under pure R light, limiting photosynthetic efficiency when water is not limiting. B light also activates cryptochrome-dependent pathways that enhance aquaporin expression, xylem water transport efficiency, and antioxidant enzyme activity, improving stress resilience by maintaining leaf hydration and reducing ROS damage under variable environmental conditions [72]. The balanced R:B ratio (RB:70:30) ensures an optimized water use efficiency by promoting stomatal activation for CO₂ fixation while maintaining water uptake and hydraulic conductivity. Excessive R light restricts stomatal conductance, while too much B light can increase transpiration beyond the plant's uptake capacity, reducing RWC. Therefore, the mixed light spectrum provides a dynamic balance between water conservation and photosynthesis, contributing to improved hydration, photosynthetic stability, and stress adaptation [73]. In conclusion, the RB:70:30 spectral composition optimizes water use efficiency by coupling B light induced stomatal activation with adequate R light driven photosynthetic energy capture. This balance is crucial for sustaining physiological homeostasis and enhancing stress tolerance in plants.

### Photosynthetic functionality decreases with increasing red light ratio, while GABA application improves it in Batavia lettuce

This study employed OJIP transient analysis as a diagnostic tool to evaluate the impact of various R:B light ratios on the efficiency of PSII in Batavia lettuce [67]. The highest F₀ values were observed under the RB:80:20 + G25 treatment. Although an elevated F₀ value is typically considered an indication of PSII reaction center inactivation or impaired excitation energy transfer from antenna complexes to reaction centers, this must be considered in the context of other fluorescence parameters. Notably, the RB:80:20 + G25 treatment also exhibited the highest F_v_/F_m_ values of all the treatments (Fig. 8), indicating that maximum PSII photochemical efficiency was not compromised. The observed increase in F₀ likely reflects adaptive modulation of energy distribution within the photosynthetic apparatus rather than photoinhibitory damage. This response may be due to GABA mediated activation of photoprotective mechanisms, such as enhanced non-photochemical quenching or reorganization of light harvesting complexes under optimal R light conditions. Therefore, interpreting F₀ alongside F_v_/F_m_ and variable fluorescence parameters (F_v_) in conjunction with the complete OJIP transient profile provides a more comprehensive and mechanistically informative assessment of photosynthetic performance under combined spectral and metabolic treatments [63, 74].

In fact, this imbalance between PSII and PSI excitation, which occurs at higher R light ratios (RB:90:10), leads to overexcitation of PSII and causes excess energy to be wasted as heat, resulting in reduced photochemical efficiency. In contrast, a more balanced R:B ratio (RB:80:20) optimizes electron flow, reduces heat loss, and improves overall photosynthetic capacity. Notably, GABA supplementation (G25) in RB:80:20 treatment further improved PSII function, reducing F₀ and DI_0_/RC values, while increasing F_v_/F_m_. These findings suggest that GABA plays a critical role in stabilizing PSII function under photoinhibitory stress conditions. However, the non-significant interaction between GABA and R:B spectral ratios for the DI₀/RC parameter suggests that GABA-mediated photoprotection depends on the spectral composition of the light and may not enhance photosynthetic performance uniformly across all light quality treatments. The elevated DI₀/RC values observed under the RB:70:30 treatment suggest that the photoprotective capacity of the applied concentration of exogenous GABA (25 µmol L⁻^1^) was exceeded by high B light exposure. Elevated B light intensifies ROS production and accelerates photosystem damage by increasing the excitation pressure on PSII reaction centers. Under such conditions, higher GABA concentrations may be necessary to sufficiently mitigate oxidative stress and stabilize dissipation processes. However, the current dosage was insufficient to produce statistically detectable interactive effects with spectral treatments [75].

At higher light ratios R, excessive R light can lead to excessive PSII depletion and QA⁻ accumulation, leading to increased DI_0_/RC. However, mid-B light portions, such as the RB:80:20 spectrum, increase PSI activity and linear electron flow, which in turn optimizes the ATP/NADPH balance that is critical for efficient photosynthesis [76]. GABA appears to modulate this process by increasing energy distribution in PSII reaction centers, reducing excess excitation energy, and reducing ROS induced damage. This is supported by changes in fluorescence parameters such as ABS/RC and TR₀/RC, where GABA treated plants showed a reduced ABS/RC ratio, indicating improved energy distribution and reaction center efficiency. In conclusion, our results emphasize that while increasing the R light ratio negatively affects photosynthetic performance, a balanced R:B ratio, especially with GABA supplementation, optimizes energy distribution, stabilizes electron transport, and reduces heat loss. These findings highlight the synergistic role of spectral combination of light and GABA in increasing photosynthetic efficiency and reducing photoinhibition, with potential applications in controlled environment agriculture to improve crop yield and quality [13, 77].

Optimal spectral balance, combined with GABA supplementation, offers a promising strategy to maximize photosynthetic performance and stress tolerance in plants, although species-specific responses and environmental factors need to be considered for adjusting light conditions in agricultural environments [78].

### Blue light dominance in RB spectra and GABA application improve lettuce appearance and flavor

Consumer preferences for lettuce quality, based on visual and taste attributes, were significantly influenced by treatment conditions. Specifically, the combination of GABA supplementation (G25) and an optimized R:B light ratio (RB:70:30 and RB:80:20) enhanced structural integrity and pigment accumulation, including chlorophyll and anthocyanins. These physiological improvements contributed to better leaf color and visual appeal, thereby boosting marketability. In contrast, the RB:90:10 spectrum, with its higher R light fraction and lower B light, resulted in reduced visual quality, likely due to insufficient B light exposure, which is crucial for stomatal regulation, relative water content, and overall plant biomass [79, 80].

Sensory evaluations demonstrated a clear impact of spectral composition and GABA supplementation on consumer acceptance. The RB:80:20 + G25 treatment received the highest taste scores, which likely reflects the higher soluble carbohydrate content measured in this treatment (Fig. 11) and is consistent with previous studies showing that sugar accumulation enhances lettuce palatability. However, a formal correlation analysis between sensory scores and carbohydrate levels was not conducted; therefore, this link remains a plausible explanation rather than a statistically confirmed relationship. Additionally, the improved taste under this treatment may also be influenced by the accumulation of secondary metabolites, such as anthocyanins and other flavonoids, and volatile organic compounds, which are known to contribute to flavor and overall palatability [81].

The optimal R:B ratio thus facilitates metabolite accumulation without triggering stress-related compounds. Conversely, the RB:70:30 + G0 treatment, lacking GABA supplementation, received the lowest consumer ratings, with over 50% of participants rejecting it. The absence of GABA likely exacerbated stress responses, negatively impacting sensory quality. These findings underline the importance of both light spectrum and nutritional supplementation in shaping lettuce quality. The synergistic effect of optimized light spectra and GABA highlights the potential for precision cultivation strategies in CEA. As CEA systems and urban agriculture continue to expand, developing protocols to optimize both visual and gustatory attributes will be essential to meet growing consumer demand for high quality fresh produce [82, 83].

## Conclusion

This study demonstrates that the combination of an optimized R:B light ratio (RB:80:20) and GABA supplementation (G25) significantly enhances the growth, performance, photosynthetic efficiency and consumer relevant quality traits of Batavia lettuce cultivated under CEA conditions. The RB:80:20 + G25 treatment notably increased shoot biomass, LA and soluble carbohydrate accumulation, while reducing energy loss (DI_0_/RC) in the electron transport chain, which indicates improved photosynthetic efficiency. While the RB:70:30 treatment stimulated greater root growth, it was associated with reduced shoot productivity. In contrast, the RB:80:20 treatment optimized the root to shoot ratio to support shoot growth and facilitate nutrient uptake. GABA supplementation provided additional benefits by enhancing the concentrations of Chl *a*, *b* and anthocyanins, thereby supporting its role in improving light utilization and plant metabolic function. Furthermore, GABA application effectively redirected plant energy allocation towards shoot development and enhanced PSII efficiency by increasing energy capture at reaction centers. Taken together, these findings establish the RB:80:20 + G25 treatment as an effective strategy for optimizing both yield and quality parameters in CEA lettuce production systems.

## Supplementary Information

Supplementary Material 1.

## Data Availability

Data will be made available on request.

## Abbreviations

GABA: γ-Aminobutyric acid
G: γ-Aminobutyric acid
CEA: Control Environment Agriculture
PAR: Photosynthetically Active Radiation
R: Red
B: Blue
Chl *a*: Chlorophyl *a*
Chl *b*: Chlorophyl *b*
RWC: Relative Water Content
VPD: Pressure Deficit
PSII: Photosystem II
PSI: Photosystem I
OJIP test: Polyphasic Chlorophyll Fluorescence Transients
F_0_: Initial Stage (Minimal Fluorescence)
F_j_: Middle Stages
F_i_: Halo Phase
F_m_: Final Peak Stage (Maximal Fluorescence)
F_m’_: Light Adapted Maximal Fluorescence
F_v_/F_m_: Maximum Efficiency Of Photosystem II
ABS/RC: Energy fluxes of light absorption per Reaction Center
TR_0_/RC: Excitation Energy Trapping Per Reaction Center
DI_0_/RC: Dissipated Energy Flux Per Reaction Center
NPQ: Non-Photochemical Quenching
QA⁻: Primary Quinone Acceptor
LEF: Linear Electron Transport
RC: PSII Reaction Center
ROS: Reactive Oxygen Species
SW: Saturated Weight
FW: Fresh Weight
DW: Dry Weight
NFT: Nutrient Film Technique
PPFD: Photosynthetic Photon Flux Density
DLI: Daily Light Integral
RH: Relative Humidity
LA: Leaf Area
ALMT: Aluminum Activated Malate Transporter
ALA: 5-Aminolevulinic Acid
ABA: Abscisic Acid
PIF: Phytochrome Interacting Factor
TCA: The Tricarboxylic Acid Cycle
