# Supplementary material for: Enhancing physiological performance and quality traits of lettuce (Lactuca sativa L. cv. 'Batavia') through red–blue light ratio and γ-aminobutyric acid interplay
Source: BMC Plant Biol. 2026 Mar 16;26:738. doi: 10.1186/s12870-026-08493-y (PMC13104395; doi:10.1186/s12870-026-08493-y)
Supplement: Supplementary file 1 — Supplementary Material 1. [file 12870_2026_8493_MOESM1_ESM.docx]

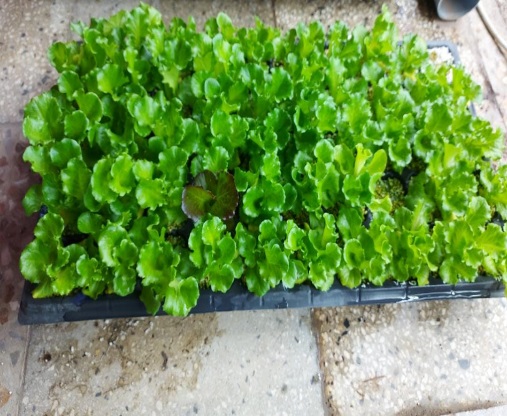


Figure 1 - Batavia lettuce seedlings ready to be transferred to the plant factory structure


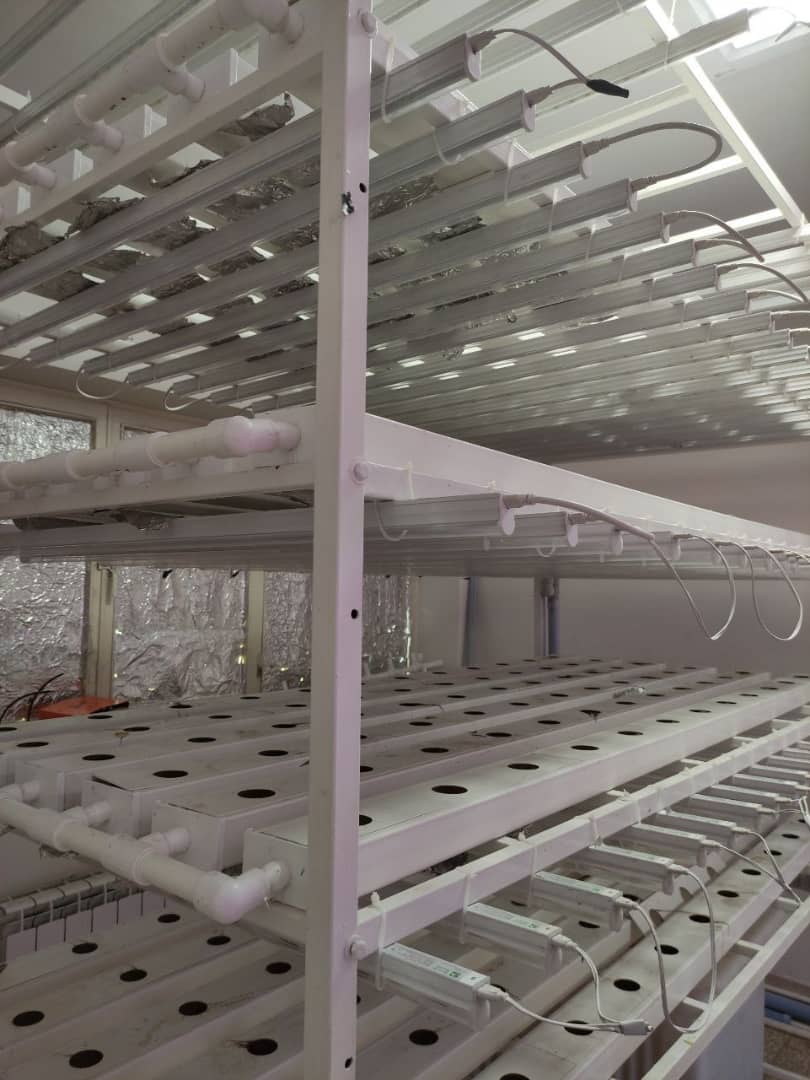


Figure 2 - Plant Factory structure before transferring seedlings to the structure

Figure 2 - Plant Factory structure before transferring seedlings to the structure


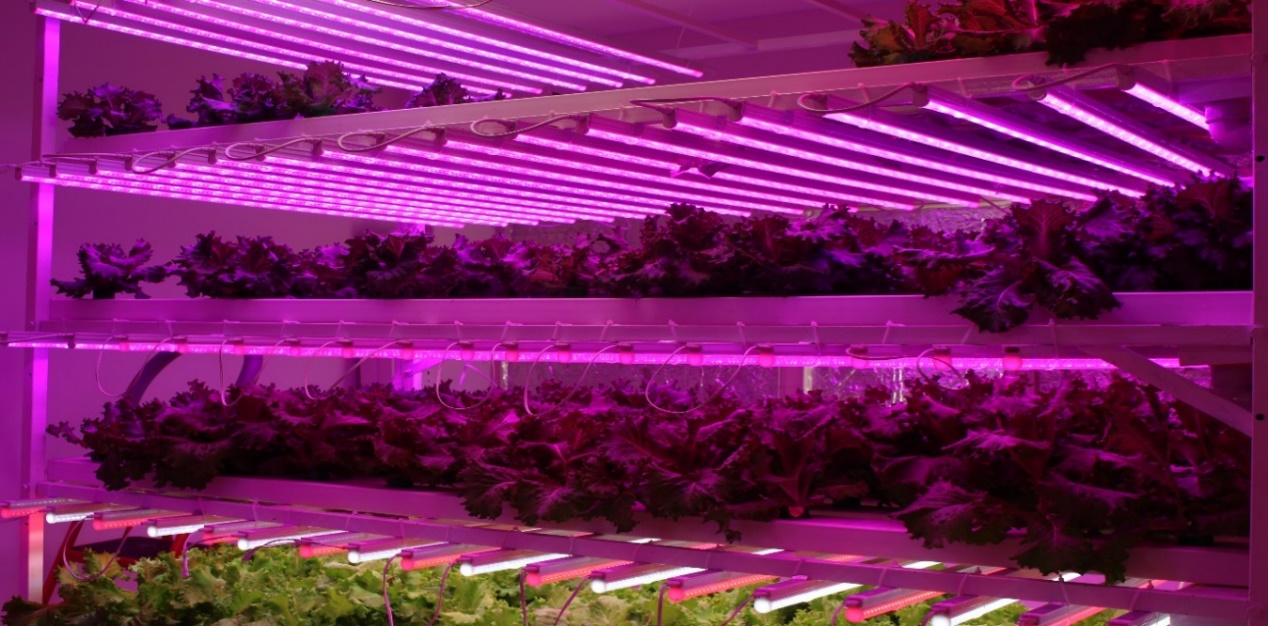


Figure 3 - Plant Factory structure after transferring seedlings to the structure and growing Batavia lettuce under artificial light in different red and blue spectra
